# Baseline Procalcitonin Levels and Their Association with Antibiotic Exposure and Clinical Outcomes in Critically Ill Patients with Suspected Bacterial Infection: A Retrospective Cohort Study

**DOI:** 10.3390/healthcare14070849

**Published:** 2026-03-27

**Authors:** I Wayan Suranadi, Ayu Dilia Febriani Wisnawa, Pontisomaya Parami, I Gusti Agung Gede Utara Hartawan, Cynthia Dewi Sinardja, Avelina Irene Djedoma

**Affiliations:** 1 Department of Anesthesiology, Pain Management and Intensive Care, Faculty of Medicine, Udayana University, Bali 80361, Indonesia; ponti@unud.ac.id (P.P.); utara_hartawan@unud.ac.id (I.G.A.G.U.H.); cynthia_dewi@unud.ac.id (C.D.S.); irebius2020@gmail.com (A.I.D.); 2 Master’s Program in Biomedical Sciences, Faculty of Medicine, Udayana University, Bali 80361, Indonesia; wisnawa.2580711020@student.unud.ac.id

**Keywords:** antimicrobial stewardship, bacterial infections, critical care, procalcitonin, intensive care unit, mortality

## Abstract

**Background/Objectives**: The excessive use of antibiotics in the intensive care unit (ICU) is driving antimicrobial resistance and poor clinical results. Therefore, the aim of this study was to assess antibiotic exposure and clinical results stratified by baseline procalcitonin (PCT) levels in critically ill ICU patients with suspected bacterial infections. **Methods**: A retrospective observational cohort study was conducted at a tertiary referral center in Bali, Indonesia. This study included adult ICU patients with suspected bacterial infections and documented baseline PCT levels. Participants were stratified into two cohorts based on a baseline PCT threshold, namely <0.5 ng/mL and ≥0.5 ng/mL. Comprehensive clinical data, antibiotic utilization, and patient results were extracted from electronic medical records. Comparative statistics and multivariable logistic regression were used to identify independent association after adjustment for relevant clinical confounders. **Results**: In this study, a total of 84 patients were analyzed. Patients in the elevated PCT group (≥0.5 ng/mL) were significantly older and had higher APACHE II scores, suggesting greater baseline illness severity at ICU admission. The median duration of antibiotic therapy was significantly longer in the elevated PCT than the normal group (8 vs. 5 days; *p* < 0.001). After adjustment for age, APACHE II score, and other relevant clinical variables, elevated baseline PCT levels remained independently associated with prolonged antibiotic exposure and in-hospital mortality. Incremental increases in PCT levels were associated with higher odds of mortality, along with advanced age and higher SOFA scores. Furthermore, the presence of multidrug-resistant (MDR) pathogens was independently associated with prolonged antibiotic use. **Conclusions**: Elevated baseline PCT levels upon ICU admission were associated with prolonged antibiotic exposure and increased in-hospital mortality. Baseline PCT levels likely reflect underlying illness severity and the magnitude of the host inflammatory response. Within antimicrobial stewardship frameworks, they should be interpreted alongside clinical assessment and severity scores rather than as a standalone determinant of antibiotic duration.

## 1. Introduction

The initial management of critically ill patients with suspected bacterial infections is the early administration of broad-spectrum antibiotics before definitive pathogen identification through microbiological cultures. In this context, treatment duration is determined empirically, guided by disease severity and the presumed source of infection [1]. However, existing antibiotic stewardship guidelines for intensive care units (ICUs) remain insufficiently precise, often leading to significant variability in clinical decision-making and a tendency toward prolonged antibiotic exposure [2]. In the absence of clear, evidence-based recommendations, clinicians often hesitate to shorten treatment courses in critically ill patients because of concerns about undertreatment and relapse risk [3].

Antibiotic stewardship in the ICU setting is further challenged by diagnostic uncertainty and the inherent complexity surrounding decisions to initiate or discontinue antimicrobial therapy. Early in the disease course, limited clinical and laboratory information may impede accurate differentiation between bacterial and nonbacterial etiologies. Concern for adverse results often leads to unnecessarily prolonged antibiotic regimens [4,5]. Previous studies have suggested that more than half of antibiotic prescriptions in hospital settings are either unnecessary or inappropriate [6]. In critically ill populations, prolonged empirical therapy is particularly common due to severe illness, overlapping clinical syndromes, and delayed microbiological confirmation. These practices are associated with increased healthcare costs, longer hospitalization, higher rates of multidrug-resistant (MDR) infections, and poorer clinical results, including increased mortality [7,8,9]. Therefore, antimicrobial resistance (AMR) is increasingly considered the most pressing global public health threat. Resistant bacterial infections are estimated to account for approximately 1.3 million direct deaths and 5 million associated deaths annually, with the greatest burden borne by low- and middle-income countries [10]. This crisis was further intensified by the increasing antibiotic use, which fostered the development of resistant pathogens. This growing challenge shows the urgent need for more precise and context-appropriate tools to guide prescription, particularly in resource-limited settings where antibiotic pressure and AMR prevalence are high [11].

Biomarkers have been widely explored as adjunctive tools to support antimicrobial stewardship (ASP) efforts, with procalcitonin (PCT) being among the most extensively investigated. Whereas C-reactive protein (CRP) is a nonspecific acute-phase protein produced by the liver in response to stimulation by interleukin (IL)-6, PCT demonstrates greater specificity for bacterial infections [12]. PCT concentrations rise rapidly within 2–6 h in response to bacterial infections and have a half-life of approximately 24 h, enabling serum levels to decrease promptly with clinical recovery. In contrast, C-reactive protein (CRP) rises within 12–24 h and may remain elevated for several days (3–7 days) despite improvement. PCT may provide advantages for dynamic monitoring of disease activity and therapeutic response due to its earlier kinetics and faster normalization [13]. In clinical practice, CRP is commonly used for general inflammatory assessment, whereas PCT has been incorporated into antibiotic stewardship algorithms, particularly for guiding discontinuation strategies. Moreover, several studies have shown that the systematic use of PCT for monitoring may also have a positive impact on the reduction in antibiotic treatment, therefore allowing a shorter stay in the ICU and lower costs per case. This will also be beneficial in combating the increase in antibiotic-resistant micro-organisms, which is mainly related to the excess use of antibiotics [14,15]. Furthermore, a ≥30% decrease in PCT levels between days 2 and 3 is considered to be an independent predictor of survival in ICU patients [16].

Several randomized controlled trials have shown that PCT-guided antibiotic discontinuation can safely reduce antibiotic exposure without adversely affecting patient results [2,17,18,19,20,21]. However, most of this evidence originates from Western healthcare settings, where baseline antibiotic consumption and AMR rates are lower [22]. Data from Southeast Asia, including Indonesia, remains limited, raising important questions regarding the generalizability of these results to regions with significantly higher antibiotic use and resistance burden. This discrepancy highlights the need to evaluate the clinical utility of baseline PCT levels within high-resistance healthcare settings.

Despite increasing interest in PCT-guided strategies, data remain scarce regarding the association between baseline PCT levels, subsequent antibiotic consumption, and clinical results among critically ill patients. A clearer understanding of this association is essential to informing antibiotic stewardship strategies designed for high-risk ICU populations and settings with elevated AMR prevalence. Therefore, the aim of this study was to evaluate the association between baseline PCT levels, antibiotic consumption patterns, and clinical results in critically ill patients with suspected bacterial infections admitted to an Indonesian ICU.

## 2. Materials and Methods

The study protocol received ethical clearance from the Udayana University Ethics Committee (Ref No: 1980/UN14.2.2.VII.14/LT/2023). Due to the retrospective observational design and the use of anonymized data, the requirement for written informed consent was waived by the ethics committee. All data were anonymized to protect patient privacy in accordance with institutional guidelines and the Declaration of Helsinki.

An observational cohort study was conducted at the ICU of Prof. Dr. I.G.N.G. Ngoerah Hospital, a tertiary referral center in Denpasar, Bali, Indonesia. The study period spanned one year, from January to December 2023. Data were systematically extracted from the integrated electronic medical records and the ICU clinical registry. This study included critically ill adult patients aged ≥18 years who exhibited clinical manifestations suggestive of bacterial infections, either at the time of ICU admission or during ICU stay. Suspected bacterial infection was operationally defined as the presence of clinical manifestations suggestive of infection (predominantly fever), accompanied by the attending intensivist’s clinical judgment supporting a bacterial etiology and initiation of empirical systemic antibiotic therapy within 24 h of ICU admission. To ensure temporal consistency and avoid reverse causality, inclusion required that patients had initiated empirical antibiotic therapy and possessed documented baseline serum PCT levels measured prior to administration of the first antibiotic dose. Microbiological confirmation was not required for study inclusion. Microbiological cultures were obtained based on clinical indication and were not performed routinely for all patients. However, culture results and antimicrobial susceptibility were further assessed when available. Multidrug resistance (MDR) was defined as acquired non-susceptibility to at least one agent in three or more antimicrobial categories according to internationally accepted consensus criteria [23]. Susceptibility testing and reporting were performed in accordance with institutional microbiology laboratory guidelines. Culture results were not used to define suspected infection but were included in multivariable analyses as potential confounders.

Several exclusion criteria were applied to ensure a homogenous study population. Pediatric patients, individuals with incomplete clinical or laboratory records, and those requiring prolonged antibiotic courses exceeding three weeks (deep-seated infections, such as endocarditis or osteomyelitis) were excluded. Furthermore, patients were excluded if confounding physiological conditions known to affect PCT levels or clinical results were present. Exclusion criteria included pregnancy or postpartum status, history of bone marrow transplantation, cardiogenic shock, Child–Pugh class C liver cirrhosis, third-degree burns, active malignancy, or severe immunocompromise. Severe immunocompromise included HIV infections with CD4+ counts below 200 cells/mm^3^, neutropenia below 500 cells/mm^3^, or chronic immunosuppressive therapy. To minimize potential survival bias and ensure comparable baseline severity, patients with a Simplified Acute Physiology Score II (SAPS-II) > 65 were excluded during eligibility screening. This criterion was applied solely for participant selection and was not incorporated into the statistical analysis.

In accordance with the institutional standard of care, serum PCT concentrations were routinely measured for all patients suspected of bacterial infections upon ICU admission. For this analysis, “baseline PCT levels” referred to the initial level obtained before the administration of the first antibiotic dose. The primary endpoint of this study was the duration of antibiotic therapy, defined as the number of days from treatment initiation to definitive cessation. Antibiotic duration was calculated as “days of therapy” (DOT), where any dose administered within 24 h accounted for one day. For the purpose of ROC analysis, prolonged antibiotic use was defined a priori as antibiotic therapy exceeding 7 days (≥7 DOT). This threshold was determined based on commonly adopted treatment durations for severe infections in critically ill patients and institutional antimicrobial stewardship benchmarks. The cutoff was specified before statistical modeling to preserve analytical validity. Secondary endpoints included in-hospital mortality rates, as well as the length of stay (LOS) in both the ICU and the general hospital wards. In-hospital mortality was defined as all-cause death occurring during the same hospitalization episode, including both ICU and post-ICU ward stay, prior to discharge. To identify potential confounders, microbiological culture results, antibiotic classes administered, and baseline markers of illness severity were recorded. The Sequential Organ Failure Assessment (SOFA) score was calculated within the first 24 h of ICU admission using the worst values for each organ system according to standard criteria to quantify the degree of organ dysfunction [24]. Additionally, the Acute Physiology and Chronic Health Evaluation II (APACHE II) score was calculated within the same timeframe to assess overall disease severity and estimated mortality risk. Both SOFA and APACHE II scores were treated as continuous variables in regression analyses to preserve statistical power and avoid arbitrary categorization.

The normality of continuous variables was assessed visually using histograms and Q-Q plots. Normally distributed data were reported as mean ± standard deviation (SD). Meanwhile, non-normally distributed variables were expressed as median with interquartile range (IQR). Categorical data were presented as absolute frequencies and percentages. Inter-group comparisons were conducted using Student’s *t*-test or the Mann–Whitney U test for continuous data and the Chi-square or Fisher’s exact test for categorical proportions, as appropriate. Receiver Operating Characteristic (ROC) curve analysis was performed to evaluate the discriminative performance of baseline PCT levels for the study outcomes. The area under the ROC curve (AUC) was calculated, and the optimal cutoff value was determined using the Youden Index, a summary measure of discriminative performance, defined as sensitivity plus specificity minus one, to identify the threshold that maximized overall test performance. Subsequently, patients were stratified into two cohorts based on this statistically derived threshold, namely the normal-PCT (n-PCT) and the elevated-PCT (e-PCT) group. Sensitivity, specificity, accuracy, and predictive values were calculated for the determined cutoff. To identify independent associations of antibiotic duration and clinical results, variables with significant associations in bivariate analysis were entered into a multivariable logistic regression model using the forward conditional method. Multicollinearity between covariates was assessed using variance inflation factor (VIF) and tolerance statistics prior to multivariable modeling. All statistical tests were two-tailed, and a *p*-value < 0.05 was defined as the threshold for statistical significance. Data analysis was performed using IBM SPSS Statistics for Windows, version 24.0 (IBM Corp., Armonk, NY, USA). Given the observational design and baseline imbalance between groups, multivariable models were interpreted as assessing associations rather than causal effects.

## 3. Results

### 3.1. Study Population and Baseline Characteristics

**Figure 1:**
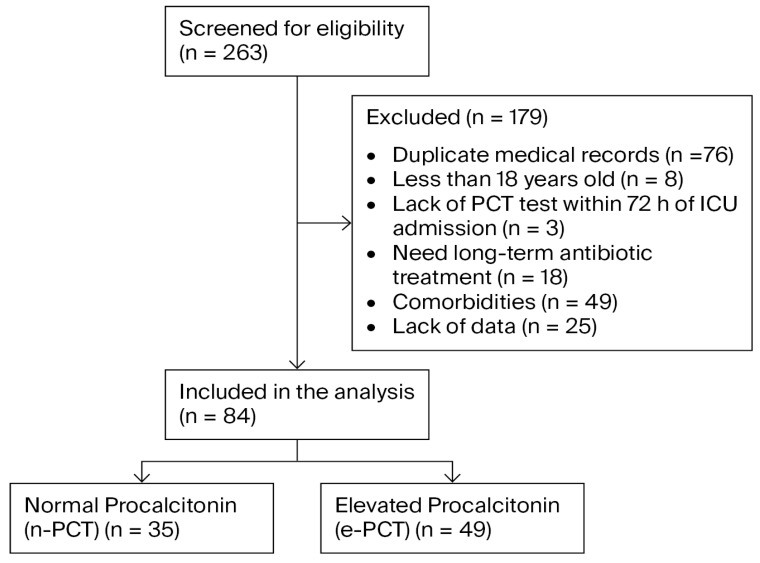
Flowchart of patient inclusion and exclusion.

A total of 263 patients with suspected bacterial infections were screened between January and December 2023 for eligibility upon admission to the ICU. Following a rigorous selection process, 179 patients were excluded for several reasons, such as duplicated records (*n* = 76), pediatric age (*n* = 8), lack of PCT measurement within 72 h of admission (*n* = 3), requirement for long-term antibiotic therapy (*n* = 18), specific exclusionary comorbidities (*n* = 49), and incomplete medical records (*n* = 25). Consequently, 84 patients met the final inclusion criteria (Figure 1).

The population was stratified into two cohorts based on a statistically derived baseline PCT threshold to optimize clinical utility for ASP. ROC curve analysis identified an optimal cutoff value of 0.5 ng/mL (Youden Index), which was subsequently used to define the groups. The normal-PCT (n-PCT) (<0.5 ng/mL) and the elevated-PCT (e-PCT) groups (≥0.5 ng/mL) comprised 35 (median PCT: 0.24 µg/L) and 49 patients (median PCT: 6.30 µg/L), respectively.

The baseline demographic and clinical characteristics are summarized in Table 1. Although the cohorts were generally comparable regarding sex and comorbidities, the e-PCT group exhibited significantly higher disease severity, characterized by older age (58.9 ± 16.8 vs. 48.5 ± 17.0 years; *p* < 0.005) and markedly higher APACHE II scores (23 [17.5–27] vs. 10 [5–20]; *p* < 0.001), indicating greater baseline illness severity. This imbalance suggests that differences in outcomes between groups may partly reflect underlying severity rather than a direct effect of PCT itself. Other inflammatory markers, such as leukocyte counts, showed no significant inter-group differences.

Furthermore, the requirements for mechanical ventilation, vasopressor support, and the prevalence of MDR pathogens were similar between the two groups. Microbiological cultures were obtained in 56 of the 84 included patients (66.7%) as culture testing was not mandatory and was performed at the discretion of the treating physicians. Overall, 40 patients (47.6% of the total cohort) had microbiologically confirmed bacterial infection, whereas the remaining cases had negative culture results or were not microbiologically tested.

### 3.2. Antibiotic Utilization Profiles

**Figure 2:**
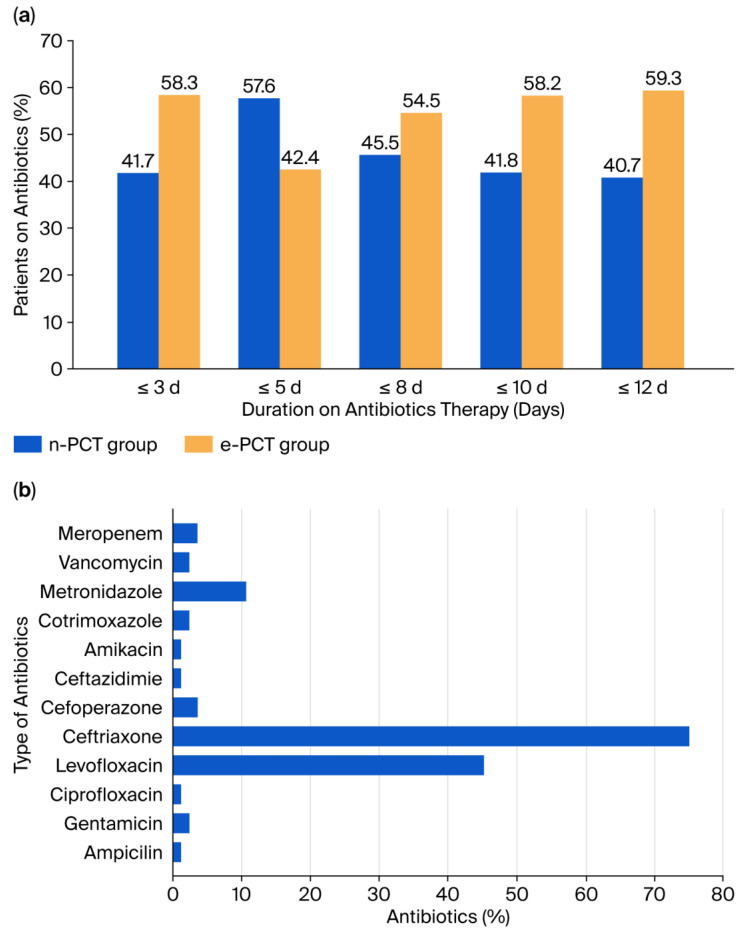
Antibiotic profiles. (**a**) Antibiotic usage duration percentages for both groups; (**b**) the distribution of antibiotics used during hospitalization.

Empirical antibiotic therapy was initiated for all study participants, comprising 35 patients in the n-PCT group and 49 in the e-PCT group within the first 24 h of ICU admission. This result coincided with the onset of suspected infections. Figure 2a shows that only 42.4% of patients in the e-PCT cohort discontinued antibiotics within five days compared with 57.6% in the n-PCT group, indicating that a larger proportion of patients in the e-PCT group continued antibiotic therapy beyond five days. These data showed that elevated baseline PCT levels were associated with a significantly more prolonged course of antimicrobial therapy.

The median duration of antibiotic treatment was significantly shorter in the n-PCT group (5 days; IQR: 5–8 days) compared with e-PCT (8 days; IQR: 5–9 days), representing a median reduction of 3 days (*p* < 0.001). In accordance with institutional protocols, all patients received empirical regimens until definitive microbiological culture results were obtained. Throughout the ICU stay, the most prevalent antibiotic agents were ceftriaxone (75%), followed by levofloxacin (45.2%) and metronidazole (10.7%). Other antibiotic classes were used in fewer than 5% of cases. In general, the prescribing pattern was characterized by heavy reliance on broad-spectrum agents (Figure 2b).

### 3.3. Discriminative Performance of Baseline PCT Level for Prolonged Antibiotic Therapy

The duration of antibiotic therapy differed significantly between groups stratified by baseline PCT levels. These findings indicate that antibiotic duration in critically ill patients is largely determined via clinical judgment and evolving patient status, and therefore, this association should not be interpreted as a direct causal effect of the biomarker.

**Figure 3:**
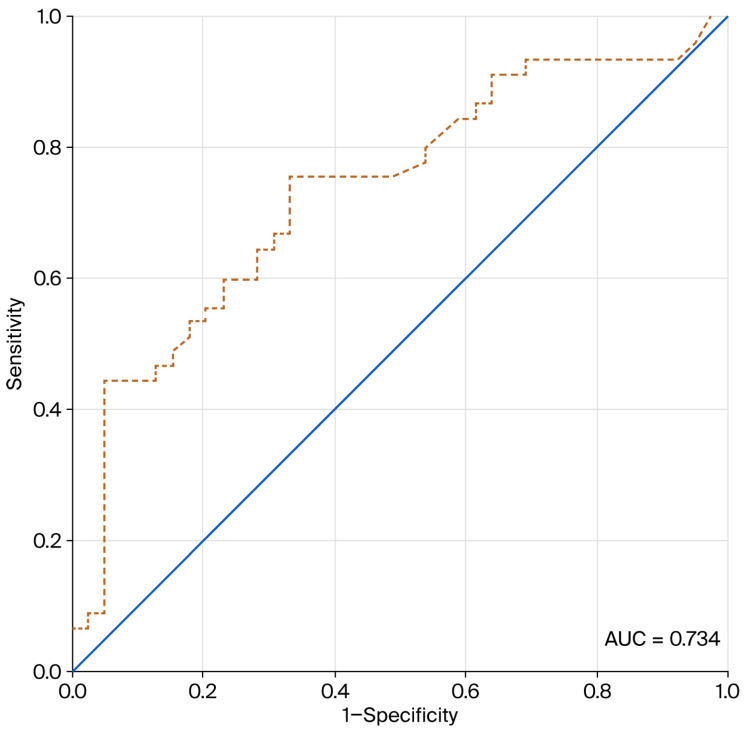
Receiver-operator characteristic area under the curve (ROC-AUC) for procalcitonin (PCT) levels as a discriminatory measure for prolonged antibiotic use.

A ROC curve analysis was carried out to assess the prognostic value of baseline PCT levels for predicting the duration of antimicrobial therapy. Baseline PCT levels demonstrated moderate discriminative performance for identifying patients who subsequently received prolonged antibiotic therapy, with an area under the curve (AUC) of 0.734 (95% CI: 0.626–0.842; *p* < 0.001) (Figure 3). This result suggested that PCT measured at ICU admission was associated with the subsequent length of antibiotic treatment.

The diagnostic characteristics of PCT are summarized in Table 2. The optimal cutoff value was identified using the Youden Index to balance sensitivity and specificity. A threshold of 0.50 ng/mL provided the best performance, producing a sensitivity of 75.6% and a specificity of 61.5% for identifying patients who required prolonged antibiotic therapy. At this cutoff, the positive predictive value (PPV) and the negative predictive value (NPV) were 69.4% and 68.6%, respectively. Evaluation of higher thresholds showed that a cutoff of 2.11 ng/mL increased specificity to 69.2% but led to a reduction in sensitivity to 66.7%. Considering this trade-off, the 0.50 ng/mL threshold was selected for subsequent analyses and for stratifying patients into normal and elevated PCT groups.

### 3.4. Multivariable Regression Analysis of Clinical Results and Antibiotic Duration

Prior to multivariable modeling, multicollinearity between covariates was assessed. No significant multicollinearity was observed between APACHE II and SOFA scores (VIF 1.167 for both; tolerance 0.857). Multivariable logistic regression models were constructed to identify independent factors associated with clinical results. Baseline PCT levels, age, and SOFA scores were confirmed as independent associations of in-hospital mortality, as shown in Table 3. APACHE II score was included in the initial multivariable model based on clinical relevance. However, it did not demonstrate independent association with mortality after adjustment for other covariates and was therefore excluded from the final model. Elevated baseline PCT showed a significant positive association with mortality risk (Wald = 4.565, *p* = 0.033). Specifically, the model produced an adjusted odds ratio (OR) of 1.088 (95% CI: 1.007–1.175), suggesting that for every 1 ng/mL increment in baseline PCT, the odds of in-hospital mortality increased by approximately 8.8%.

Age maintained independent prognostic significance (Wald = 7.209, *p* = 0.007, OR = 1.055, 95% CI: 1.014–1.096), with each additional year corresponding to a 5.5% increase in mortality risk. Higher SOFA scores at the time of ICU admission were also strongly correlated with adverse results (Wald = 7.666, *p* = 0.006, OR = 1.236, 95% CI: 1.064–1.435). A one-point increase in the SOFA score was associated with a 23.6% higher risk of death.

Regarding the duration of antimicrobial therapy, the multivariable analysis summarized in Table 4 identified both baseline PCT concentrations and the presence of MDR pathogens as significant independent predictors of prolonged treatment. Higher initial PCT levels were associated with an increased probability of extended antibiotic courses (Wald = 4.167, * *p* = 0.041, OR = 1.085, 95% CI: 1.003–1.174), suggesting that an 8.5% increase in the odds of prolonged therapy occurred with every 1 ng/mL rise in PCT.

Detection of MDR pathogens was independently associated with prolonged antibiotic duration (Wald = 3.876, *p* = 0.049), with an adjusted OR of 4.153 (95% CI: 1.006–17.140). The wide confidence interval indicates limited precision of the estimate and likely reflects the relatively small number of MDR cases in the cohort. Overall, these findings suggest that both baseline PCT levels and microbiological resistance profiles are associated with antibiotic utilization patterns in the ICU. Collectively, these data showed the combined prognostic contribution of initial biomarker levels and microbiological resistance profiles on stewardship-related results in the ICU.

## 4. Discussion

Distinguishing infectious from non-infectious causes of systemic inflammation at ICU admission remains challenging in critically ill patients and often leads to early empirical antibiotic initiation and potential overuse [25]. Although microbiological cultures were obtained in a subset of patients, the study was not designed to classify definitive bacterial versus non-bacterial infections. These findings should therefore be interpreted within the context of a clinically defined suspected infection cohort. In this setting, baseline procalcitonin (PCT) was evaluated as an adjunctive biomarker to support early risk stratification and to explore its relationship with antibiotic utilization and clinical outcomes.

Within this real-world suspected infection cohort, baseline PCT demonstrated acceptable discriminative performance (AUC 0.734) for identifying patients with prolonged antibiotic exposure and adverse clinical outcomes. A baseline PCT threshold of 0.50 ng/mL provided early adjunctive risk-stratification information, consistent with thresholds commonly used in previous PCT-guided antimicrobial strategies. Concentrations below this threshold have been associated with lower infection severity and reduced antibiotic utilization in critically ill patients. This threshold has been commonly interpreted as suggestive of a lower probability of clinically significant systemic bacterial infection and may therefore reflect a less pronounced systemic inflammatory response [2,19,26]. In addition, patients with lower baseline PCT levels in our cohort had an approximately three-day shorter duration of antibiotic therapy. This finding is consistent with a meta-analysis of randomized controlled trials showing that PCT-guided antimicrobial strategies reduce antibiotic exposure by approximately two days in critically ill patients [27]. Importantly, this study reflects routine clinical practice rather than an interventional PCT-guided protocol. Decisions regarding treatment continuation or discontinuation are influenced by multiple factors, including perceived illness severity, microbiological findings, treatment response, and clinician risk tolerance. Consequently, the observed relationship between higher PCT levels and longer antibiotic therapy may partly reflect physicians’ risk perception in patients presenting with greater inflammatory burden.

This study suggests that baseline PCT levels are associated with antimicrobial consumption and in-hospital mortality. Although multivariable adjustment was performed, baseline severity differences may have influenced these relationships. Our analysis suggested that a baseline PCT threshold around 0.50 ng/mL was associated with differences in antibiotic exposure and clinical outcomes in this cohort. However, this threshold was derived from internal ROC analysis and should be interpreted as exploratory rather than a definitive clinical decision boundary, providing adjunctive risk-stratification information that may complement, but not replace, clinical assessment and established severity scores. In an era dominated by escalating AMR, these results may provide preliminary contextual data that could inform future antimicrobial stewardship strategies in Southeast Asian ICU settings where diagnostic resources are often limited.

The determination of a 0.50 ng/mL optimal cutoff is fundamentally consistent with the biological role of PCT as a proxy for systemic bacterial load. This specific threshold achieved a pragmatic equilibrium between sensitivity (75.6%) and specificity (61.5%). Although landmark investigations, such as the ProRATA and ProHOSP trials, have scrutinized various thresholds for terminating therapy, this study’s observations show a key result. Within the high-acuity environment, this threshold may help contextualize the likelihood of substantial bacterial inflammation from localized or non-infectious inflammatory triggers [21,26]. Shifting this threshold to 2.11 ng/mL improved specificity to approximately 70%. This result suggests that exceptionally high baseline PCT might function as an early clinical “red flag” for complex infectious processes resistant to abbreviated antibiotic regimens.

Notwithstanding its acceptable discriminative performance, the moderate specificity and predictive values indicate that baseline PCT alone is insufficient to definitively determine which patients require prolonged antimicrobial therapy. This observation is consistent with previous studies demonstrating that procalcitonin should not be used as a standalone determinant for antibiotic decisions but rather interpreted alongside clinical and microbiological findings [26]. In critically ill populations, antibiotic duration is influenced by multiple dynamic factors, including clinical trajectory, microbiological confirmation, adequacy of source control, and progression of organ dysfunction. Accordingly, baseline PCT should be regarded as an adjunctive risk-stratification biomarker within antimicrobial stewardship frameworks rather than a deterministic tool for guiding antibiotic duration.

In this cohort, pulmonary infection was the most frequent suspected source of infection. Consequently, empirical antimicrobial therapy was primarily guided by clinical assessment at presentation rather than microbiological confirmation. This approach reflects standard practice for critically ill patients, where rapid antibiotic initiation is prioritized. Metronidazole was administered to approximately 10% of the cohort, specifically when anaerobic involvement was clinically suspected or the infection source was unclear. The lack of isolated anaerobic pathogens in these results should be interpreted with caution. Empirical antibiotics may have preceded microbiological sampling, and anaerobic cultures were not systematically performed. Moreover, anaerobic organisms are technically challenging to recover due to their oxygen sensitivity and specific transport requirements. Consequently, culture-based microbiological results may underestimate the contribution of anaerobic pathogens during initial empirical treatment decision-making [28].

A significant result in the analysis is the profound impact of MDR organisms on treatment trajectories. In the multivariable model, MDR infection was associated with a more than fourfold increased likelihood of prolonged antibiotic therapy (OR 4.153; 95% CI: 1.006–17.140). This result shows a critical clinical dilemma as while an elevated baseline procalcitonin (PCT) reflects greater inflammatory burden, it does not provide information regarding antimicrobial resistance profiles. This finding aligns with previous reports indicating that infections caused by MDR pathogens frequently lead to prolonged antimicrobial exposure and delayed de-escalation due to concerns regarding treatment failure and limited therapeutic options [26,29]. In our cohort, the frequent use of broad-spectrum agents, particularly ceftriaxone (75%), suggests a “defensive” prescribing pattern in high-resistance setting. In such contexts, clinicians may maintain extended antimicrobial coverage until clear clinical stability is achieved. This gap between biomarker signaling and antibiotic discontinuation underscores the importance of antimicrobial stewardship. Integrating baseline PCT with rapid molecular diagnostics for resistance may help facilitate earlier and safer de-escalation, particularly in patients with low inflammatory burden even in high-AMR environments.

Beyond antibiotic exposure, baseline PCT levels were associated with in-hospital mortality (OR 1.088 per 1 ng/mL rise) after adjustment for age, severity status, and other relevant parameters. Consequently, PCT captures a dimension of the host’s inflammatory response to bacterial insult that is not fully represented by physiological organ dysfunction scores. A prior study also reported associations between elevated procalcitonin levels and increased mortality risk, suggesting that PCT may reflect the magnitude of the systemic inflammatory response to severe bacterial infection [30]. The 8.8% increase in mortality risk for every 1 ng/mL rise in PCT shows that baseline levels should be viewed not just as a diagnostic “yes/no” for infections but as a continuous scale of inflammatory severity. The “dose–response” relationship allows for more precise risk stratification during the “golden hours” of ICU admission. However, a substantial baseline imbalance was present between PCT groups, particularly in age and APACHE II scores, indicating that patients with higher PCT were also more severely ill at admission. In this context, baseline PCT likely functions primarily as a surrogate marker of disease severity and clinician-perceived risk rather than a direct determinant of treatment duration or outcome. Although multivariable adjustment attenuated this effect and PCT remained statistically associated with outcomes, residual confounding by severity cannot be excluded due to the modest sample size and the correlation between inflammatory biomarkers and physiological scores. Baseline PCT should consequently not be interpreted as an independent stewardship-guiding trigger but rather as an adjunctive indicator that reflects the overall inflammatory burden and should be interpreted together with clinical severity indices such as SOFA and APACHE II.

This study provides region-specific data from Indonesia, a region where a high infectious disease burden often leads to excessive antibiotic pressure. The median reduction of three days in antibiotic duration in the n-PCT group (5 vs. 8 days) is clinically significant. The 0.50 ng/mL threshold may assist antimicrobial stewardship teams in contextual decision-making when interpreted alongside clinical severity and microbiological data. This potentially reduces the selection pressure for future MDR strains without compromising patient safety. These findings, consistent with evidence from randomized controlled trials and meta-analyses, have demonstrated that procalcitonin-guided strategies reduce antibiotic exposure without increasing mortality or treatment failure, supporting the safety of this approach across diverse clinical settings [30].

This study depended on a static baseline PCT value. Given the retrospective design and routine practice in our setting, this study intentionally focused on baseline PCT levels as a single baseline measurement to evaluate its association with antibiotic duration and clinical outcomes in routine ICU practice, where serial monitoring is not consistently available. Our findings indicate that even a single baseline value is associated with antimicrobial exposure and mortality. Nevertheless, recent evidence suggests that PCT kinetics over the first 48–72 h may better reflect treatment response and antibiotic failure. Future prospective studies in the Indonesian context should investigate whether serial measurements provide incremental clinical value and may further optimize antibiotic duration compared with baseline assessment alone.

Several limitations require consideration as the retrospective design at a single center may limit the external validity and generalizability of the findings to other healthcare settings with different patient populations, antimicrobial stewardship practices, and antimicrobial resistance patterns. Second, there was a significant imbalance in baseline severity between PCT groups, particularly in age and APACHE II scores. Although multivariable adjustment was performed, residual confounding and confounding by indication remain likely. Third, the sample size limited the ability to perform propensity matching or stratified analyses by severity, which would be necessary to more definitively assess whether PCT provides prognostic information beyond established severity scores. The relatively limited number of outcome events in relation to the number of predictors included in the regression models may also increase the risk of model instability and imprecise effect estimates. The findings should therefore be interpreted as exploratory and hypothesis-generating and should be validated in larger prospective multi-center studies. Despite these limitations, the study provides region-specific evidence from a high antimicrobial resistance setting, which may contribute valuable contextual data for antimicrobial stewardship strategies in similar environments.

## 5. Conclusions

In conclusion, this study shows that elevated baseline PCT levels measured at ICU admission were associated with prolonged antibiotic exposure and higher in-hospital mortality among critically ill patients. ROC analysis suggested an exploratory cutoff value of approximately 0.50 ng/mL, demonstrating moderate discriminatory performance within this cohort. Patients presenting above this threshold show a greater tendency to receive prolonged antibiotic therapy and experience poorer survival results. Furthermore, the presence of MDR pathogens is an independent determinant of treatment complexity, remaining associated with prolonged antimicrobial use regardless of baseline PCT levels.

The results of this study show that incorporating baseline PCT levels is associated with illness severity, antibiotic exposure, and mortality. Their clinical value (0.50) may provide supportive information for early risk stratification among critically ill patients with suspected bacterial infection rather than a stand-alone stewardship-guiding biomarker when interpreted alongside clinical severity scores and microbiological findings. Future prospective studies should assess whether stewardship strategies guided by serial PCT measurements can further optimize antibiotic duration and improve clinical results in critically ill patients in the Indonesian ICU setting.

## Figures and Tables

**Table 1:** Baseline characteristics of the study participants.

Characteristics	Overall	n-PCT Group	e-PCT Group	*p*-Value
	(*n* = 84)	(*n* = 35)	(*n* = 49)	
Age (mean ± SD), yr	54.5 ± 17.6	48.5 ± 17	58.9 ± 16.8	0.005 ^a^ *
Sex (*n*, %), male	55 (65.5%)	22 (62.9)	33 (67.3)	0.670 ^b^
Comorbidities (*n*, %), yes	57 (67.9)	22 (62.9)	35 (71.4)	0.407 ^b^
Diabetes mellitus	15 (17.9)	9 (25.7)	6 (12.2)	0.112 ^b^
Cardiovascular disease	25 (29.8)	8 (22.9)	17 (34.7)	0.242 ^b^
Chronic lung disease	13 (15.5)	5 (14.3)	8 (16.3)	0.799 ^b^
Chronic kidney disease	7 (8.3)	1 (2.9)	6 (12.2)	0.230 ^c^
Liver disease	1 (1.2)	0 (0)	1 (2)	1.000 ^c^
Malignancy	8 (9.5)	4 (11.4)	4 (8.2)	0.714 ^c^
Blood pressure (median, IQR), mmHg				
Systolic	105.5 (90–130)	110 (91–134)	102 (89–130)	0.393 ^a^
Diastolic	69.5 (60–81.8)	70 (60–90)	69 (60–79)	0.453 ^a^
Temperature (median, IQR), °C	38 (37.8–38.8)	38 (37.8–38.6)	38 (37.8–38.9)	0.848 ^a^
Site of infection (*n*, %)				
Pulmonary	52 (61.9)	19 (54.3)	33 (67.3)	0.514 ^a^
Intraabdominal	5 (6)	0 (0)	5 (10.2)	
Urinary tract	1 (1.2)	0 (0)	1 (2)	
Others	3 (3.6)	2 (5.7)	1 (2)	
Bacterial pathogen identified (*n*, %), Gram-negative	26 (31)	10 (28.6)	16 (32.7)	0.453 ^a^
Gram positive pathogens				
Methicillin-resistant *S. aureus*	1 (1.2)	1 (2.9)	0 (0)	
Methicillin-susceptible *S. aureus*	4 (4.8)	1 (2.9)	3 (6.1)	
Coagulase-negative staphylococci	4 (4.8)	1 (2.9)	3 (6.1)	
*Viridans streptococcus*	1 (1.2)	1 (2.9)	0 (0)	
Gram negative pathogens				
*Acinetobacter baumannii*	9 (10.7)	2 (5.7)	7 (14.3)	
*Enterobacter* spp.	1 (1.2)	0 (0)	1 (2)	
*Escherichia coli*	3 (3.6)	0 (0)	3 (6.1)	
*Klebsiella pneumoniae*	9 (10.7)	6 (17.1)	3 (6.1)	
*Pseudomonas aeruginosa*	4 (4.8)	1 (2.9)	3 (6.1)	
Others	3 (3.6)	2 (5.7)	1 (2)	
Antibiotic (*n*, %), ≥2		13 (37.1)	22 (44.9)	0.477 ^b^
MDR pathogens (*n*, %), yes	18 (21.4)	5 (14.3)	13 (26.5)	0.278 ^a^
Chest x-ray findings (*n*, %)				
Unilateral consolidations	20 (23.8)	8 (22.9)	12 (24.5)	0.973 ^a^
Bilateral consolidations	39 (46.4)	17 (48.6)	22 (44.9)	
Normal	25 (29.8)	10 (28.6)	15 (30.6)	
Laboratory findings (median, IQR)				
White blood cells, ×10^3^/µL	15.8 (11–20.1)	15.6 (10.6–18)	14.9 (11.3–20.9)	0.618 ^a^
Procalcitonin, ng/mL	2.1 (0.3–8.5)	0.24 (0.11–0.40)	6.30 (2.95–15.89)	<0.001 ^a^ ***
Clinical status on ICU admission (*n*, %)				
Need for MV	30 (35.7)	15 (42.9)	15 (30.6)	0.912 ^a^
Need for vasopressor support	22 (26.2)	5 (14.3)	17 (34.7)	
Need both support (MV and vasopressor)	32 (38.1)	15 (42.9)	17 (34.7)	
Severity of illness (*n*, %)				
APACHE II score	19.5 (9.3–27)	10 (5–20)	23 (17.5–27)	<0.001 ^a^ ***
SOFA score	6 (3.3–10)	5 (3–10)	7 (4–11)	0.259 ^a^

Data are presented as mean ± standard deviation (SD) for normally distributed variables, median with interquartile range (IQR) for non-normally distributed variables, and number (percentage) for categorical variables. *p*-values represent comparisons between the normal-PCT (*n* = 35) and elevated-PCT (*n* = 49) groups. Asterisk (*) indicates statistically significant *p*-values. * *p* < 0.05, *** *p* < 0.001. ^a^ *p* values were calculated using the Mann–Whitney u-test. ^b^ *p* values were calculated using the chi-square test. ^c^ *p* values were calculated using the fisher’s exact test. Abbreviation: ICU, intensive care unit; PCT, procalcitonin; spp, species; APACHE, Acute Physiology and Chronic Health Evaluation; MDR, multidrug-resistant; MV, mechanical ventilation.

**Table 2:** Predictive performance of baseline procalcitonin levels for prolonged antibiotic duration.

Procalcitonin (ng/mL)	Value	95% CI	*p*-Value
Area Under the Curve (AUC)	0.734	0.626–0.842	<0.001 ***
Optimal Cut-off (ng/mL)	0.5		
Sensitivity (%)	75.6	60.5–87.1	
Specificity (%)	61.5	44.6–76.6	
Positive Predictive Value/PPV (%)	69.4	54.6–81.7	
Negative Predictive Value/NPV (%)	68.6	50.7–83.1	

Asterisk (*) indicates statistically significant *p*-values. *** *p* < 0.001. Abbreviation: 95% CI, 95% confidence interval.

**Table 3:** Factors independently associated with in-hospital mortality.

Dependent Variable: In-Hospital Mortality	Wald	*p*-Value	OR	95% CI
Procalcitonin (initial level, ng/mL)	4.565	0.033 *	1.088	1.007–1.175
Age, yr	7.209	0.007 *	1.055	1.014–1.096
SOFA score	7.666	0.006 *	1.236	1.064–1.435

Asterisk (*) indicates statistically significant *p*-values. * *p* < 0.05. Abbreviation: 95% CI, 95% confidence interval; yr, years.

**Table 4:** Factors independently associated with the duration of antibiotic use.

Dependent Variable: Duration of Antibiotic Use	Wald	*p*-Value	OR	95% CI
Procalcitonin (initial level, ng/mL)	4.167	0.041 *	1.085	1.003–1.174
MDR pathogens (ref cat-no)	3.876	0.049 *	4.153	1.006–17.140

Asterisk (*) indicates statistically significant *p*-values. * *p* < 0.05. Abbreviation: 95% CI, 95% confidence interval; ref cat, reference category.

## Data Availability

The original contributions presented in this study are included in the article. Further inquiries can be directed to the corresponding author.

## Abbreviations

The following abbreviations are used in this manuscript:

ICU	Intensive Care Unit
PCT	Procalcitonin
n-PCT	Normal Procalcitonin
e-PCT	Elevated Procalcitonin
APACHE	Acute Physiology And Chronic Health Evaluation
MDR	Multidrug-Resistant
AMR	Antimicrobial Resistance
SAPS	Simplified Acute Physiology Score
LOS	Length of Stay
DOS	Days of Therapy
ASP	Antimicrobial Stewardship Program
ROC	Receiver Operating Characteristic
AUC	Area Under the Curve
IQR	Interquartile Range
MV	Mechanical Ventilation
